# Intravenous Sodium Ferric Gluconate Complex for Hospitalized Pediatric Patients with Iron Deficiency Anemia

**DOI:** 10.3390/children12020189

**Published:** 2025-02-05

**Authors:** Felicia White, Noelle Polakowski, Elena Merlington, Mara Leimanis, Brooke Dudick, Jessica Parker, Ashley Jousma, Alexander Loji, Jeffrey Hanson, Monica Arney, Brian Boville

**Affiliations:** 1 College of Human Medicine, Michigan State University, Grand Rapids, MI 49503, USA; felicia.white@corewellhealth.org (F.W.); noelle.l.polakowski@hitchcock.org (N.P.); elena.ybarra2@corewellhealth.org (E.M.); mara.leimanis@corewellhealth.org (M.L.); alexander.loji@duke.edu (A.L.); jeffrey.hanson@aah.org (J.H.); mml3mg@uvahealth.org (M.A.); 2 Department of Pediatrics, Corewell Health Helen DeVos Children’s Hospital, Grand Rapids, MI 49503, USA; 3 Division of Pediatric Critical Care & Sedation, Corewell Health Helen DeVos Children’s Hospital, Grand Rapids, MI 49503, USA; 4 Department of Bioinformatics, Corewell Health Health DeVos Children’s Hospital, Grand Rapids, MI 49503, USA; brooke.dudick@corewellhealth.org (B.D.); jessica.parker2@corewellhealth.org (J.P.); 5 Department of Quality, Safety, & Experience, Corewell Health Helen DeVos Children’s Hospital, Grand Rapids, MI 49503, USA; ashley.jousma@corewellhealth.org

**Keywords:** iron deficiency, anemia, intravenous iron, red blood cell transfusion, sodium ferric gluconate complex, pediatric

## Abstract

**Background/Objectives:** Iron deficiency anemia is common in the pediatric population. Red blood cell transfusions, a common acute treatment, pose well-recognized risks including lung injury, circulatory overload, and immune dysfunction. Intravenous iron, specifically sodium ferric gluconate complex (SFGC), is a potential alternative, however investigation on its use in hospitalized children is lacking. This study aims to describe the physiologic response via change in hematologic values to cumulative dose of SFGC, investigate the effect of cumulative dosing on the amount of RBC transfusions received, and comment on its safety. **Methods**: This is a retrospective investigation of pediatric patients with iron deficiency who received SFGC during their admission to the Helen DeVos Children’s Hospital between 2016 and 2018 (N = 85). **Results:** A total of 258 doses of intravenous SFGC were provided to 85 patients. The average pre-treatment serum hemoglobin was 8.73 ± 1.33, and 7 days post-treatment this increased to 10.41 ± 1.43. Mean corpuscular volume, ferritin, serum ion, total iron binding capacity, reticulocyte percentage, and reticulocyte hemoglobin all increased 7 days post-treatment, as would be suspected, but without any statistically significant difference between hematologic outcomes and cumulative dose of SFGC. Our study did not reveal any correlation between the cumulative dose of SFGC administered and the amount of RBC transfusions received. Only one adverse event was recorded. **Conclusions:** Our results complement the trend of increased use and emerging evidence of favorable safety profiles of IV iron in the pediatric population. This descriptive investigation revealed that administering higher cumulative doses of SFGC provided no further benefits in terms of hematologic response or RBC transfusion administration.

## 1. Introduction

Anemia is a common comorbid condition for pediatric patients, with an incidence up to 74% in the pediatric intensive care unit [1]. The most common cause of anemia in the critical care setting is iron deficiency [2,3]. Iron deficiency is a common comorbid condition in children who suffer from disorders involving increased blood loss, bone marrow suppression in anemia of chronic disease, and malabsorptive states [4,5,6].

Red blood cell transfusions are a common acute treatment for anemia in critically ill children. There are multiple associated risks including transfusion-associated lung injury, transfusion-associated circulatory overload, and immune dysfunction which are prevalent in this population [1,7,8,9,10,11,12]. Given the increased morbidity associated with transfusions, consensus recommendations have called for a more restrictive transfusion strategy [7]. Efforts to investigate the use of supportive therapies such as iron replacement and erythropoiesis-stimulating agents to reduce the transfusion burden are needed.

While there are available data related to utilizing intravenous (IV) iron therapy as a treatment for specific pediatric illness such as inflammatory bowel disease [13,14] and hemodialysis patients [15,16], there are little data addressing the utilization of IV iron therapy in a hospitalized child. A recent 2024 study investigated the use of IV iron sucrose in the critically ill pediatric population, which demonstrated efficacy and safety of this therapy [17]. However, there have been minimal published investigations of the other rapid release IV iron formulations, such as the sodium ferric gluconate complex, amongst this patient population.

**Figure 1:**
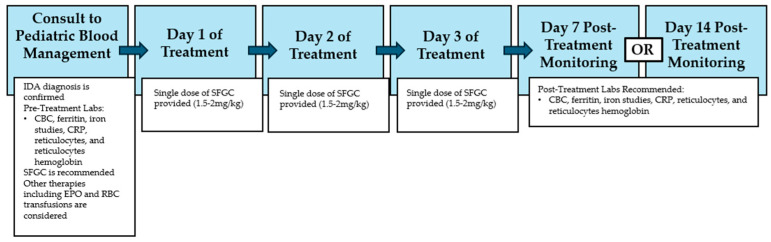
Timeline for patients who received SFGC at HDVCH. Beginning with admission and consultation with the pediatric blood management team, to receiving SFGC, to post-treatment monitoring; this figure provides the timeline that patients experienced. HDVCH: Helen DeVos Children’s Hospital, SFGC: sodium ferric gluconate complex, IDA: iron deficiency anemia, CBC: complete blood count, CRP: c-reactive protein, EPO: epoetin, RBC: red blood cell.

Intravenous sodium ferric gluconate complex (SFGC), along with other newer carbohydrate iron formulations have been demonstrated to be effective in improving iron deficiency with a low rate of severe adverse drug reactions and barriers to treatment [18,19]. Helen DeVos Children’s Hospital (HDVCH) is a large tertiary center in Michigan that commonly utilizes SFGC. A standardized clinical workflow for hospitalized pediatric patients with iron deficiency anemia at this institution involves an initial consultation under the Pediatric Blood Management Program (PBMP). Figure 1 demonstrates the timeline for a patient with confirmed iron deficiency anemia involved in this experience. The standard of care at this institution is administration of an SFGC 1.5–2 mg/kg dose once daily for three consecutive days, with monitoring serum laboratory recommendations provided for either 7 days post-treatment or 14 days post-treatment. Prior work at our institution has shown both safety and success in the rapid correction of iron deficiency anemia utilizing this dosing protocol. Further explanation of the PBMP and dosing regimen of SFGC was elucidated by previously published work done at this institution by Hassan et al. [20].

The primary objective of this retrospective study was to offer a description of the physiologic response via change in hematologic values to cumulative dose of SFGC between 7 and 14 days post-treatment. The primary outcome variable was hemoglobin, and the secondary outcome variables were MCV, ferritin, serum iron, TIBC, CRP, reticulocyte count, and reticulocyte hemoglobin at 7 and 14 days post-treatment. The secondary objective was to investigate the correlation between cumulative dose of SFGC and the amount of RBC transfused. The final objective was to comment on the safety profile of SFGC experienced by our patients. We hypothesized that our institutional standard of a three-dose series of SFGC is enough to produce an adequate physiologic response, decrease the need for RBC transfusions, and demonstrate safety.

## 2. Materials and Methods

### 2.1. Study Site and Patient Population

Following institutional review board (IRB) approval (#2018-464), we conducted a retrospective medical records review of all pediatric patients, less than 18 years old, at Helen DeVos Children’s Hospital between 1 January 2016 and 31 December 2018 who received an infusion of SFGC for treatment of iron deficiency as part of their care while hospitalized. Consent was waived by the IRB due to the minimal risk, retrospective nature, and inclusion of all patients in the study. Iron deficiency was defined as iron saturation <20%, serum iron <30 µ/mL, or ferritin <50 µg/mL [21]. Indications for SFGC included iron deficiency secondary to acute blood loss, chronic blood loss, malabsorption/nutritional deficiency, or bone marrow suppression. Indications were reported based on the primary reason for administration of SFGC according to the documented medical decision-making process at the time of treatment and review of the clinical scenario by data extractors.

### 2.2. Data Extraction and Quality Control

Patient medical charts were collected by HDVCH pharmacy from Cerner and Epic medical records. The data were extracted by three of the co-authors (F.W., N.P., E.M.). Secondary review was performed on 10% of the total data collected by a fourth co-author (B.B.), who found a 0.57% error rate. Indication for infusion was reviewed by primary authors (F.W., B.B.) and aggregated by consensus into a categorical variable (acute blood loss, chronic blood loss, malabsorption/nutritional deficiency, bone marrow suppression).

### 2.3. Data Variables and Hematological Timepoints

Patient descriptors collected included continuous variables such as age, weight, dates, and times related to admission and treatment initiation, as well as categorical variables such as sex assigned at birth, race, admission diagnosis, indications for SFGC, and pediatric intensive care unit (PICU) versus general pediatrics hospital admission. We collected Pediatric Risk of Mortality III (PRISM III) scores for patients admitted to the PICU utilizing the Collaborative Pediatric Critical Care Research Network (CPCCRN) manual calculator, which incorporates prognostically important physiologic data within the interval from 2 h prior to PICU admission through to 4 h after admission [22]. PRISM III is a widely used and well validated physiology-based scoring methodology which quantifies critical illness and mortality risk for pediatrics. Scores range from 0 to 74, with higher scores indicating a greater risk of mortality [22,23,24].

Additional numerical variables collected included the total number of SFGC doses received, total cumulative dose of SFGC received, volume of packed red blood cells (RBCs) received, and total blood loss during the admission (mL), as well as binary variables such as recombinant erythropoietin (epoetin) administration within 48 h before or after SFGC infusions, and adverse drug reactions. Total cumulative dose of SFGC was obtained through data extraction and calculated as the total milligrams received throughout the entire hospitalization for each patient. Outcome variables extracted included continuous variables such as serum hemoglobin, mean corpuscular volume (MCV), ferritin, serum iron, total iron binding capacity (TIBC), c-reactive protein (CRP), reticulocyte count, and reticulocyte hemoglobin. For all the outcome variables listed above, multiple values were recorded for each patient. These included a measure at the time of admission, a pre-treatment measure (defined as 24 h or less before an infusion of SFGC was administered), a post-treatment measure closest to day 7 while within 5–10 days of the last infusion, and a post-treatment measure closest to day 14 while within 11–17 days of the last infusion. The post-treatment measurement time frames were chosen based on the demonstration that in moderate or severe anemia, defined as hemoglobin less than 9 g/dL, a reticulocyte response peaks 7 days after initiation of treatment, [25,26] and it can be expected that hemoglobin would continue to rise even after the maximum reticulocyte response is achieved. 

### 2.4. Statistical Analysis 

The primary outcome variable for this study is the correlation between hemoglobin rise and the cumulative dose of SFGC. An a priori power analysis was conducted using G*Power to determine the minimum sample size needed to detect a statistically significant association. Assuming that r = 0.5, with alpha = 0.05, and beta = 0.20, we should be able to detect a statistically significant correlation with a sample size of n = 29 using a Pearson’s correlation coefficient.

Quantitative data were expressed as mean ± standard deviation or median [25th, 75th percentile], depending on normality. Normality was assessed by looking at a histogram, using a Shapiro–Wilk *p*-value and reviewing the skewness and kurtosis of the data points. Categorical data were expressed as count (%). Patient outcomes and characteristics were described with the exposure variable of SFGC as a categorical variable. Descriptive hematologic values were presented as a whole sample, not considering a categorical or continuous variable. Quantitative descriptors and outcomes were compared between three dose groups using Kruskal–Wallis tests, as none of the variables met normality assumptions. Categorical descriptors and outcomes were compared between three dose groups using Chi-square, or if more than 20% of the expected cell counts were less than five, a Fisher’s exact test was used. Additionally, the exposure variable of SFGC was utilized as a continuous variable using Pearson’s correlation coefficient to detect correlations amongst various dependent variables. All statistical significance was assessed at *p* < 0.05. All statistical analyses for this project were completed using SAS Enterprise Guide Software, Version 7.1, SAS Institute Inc., Cary, NC, USA.

## 3. Results

### 3.1. Study Population

**Figure 2:**
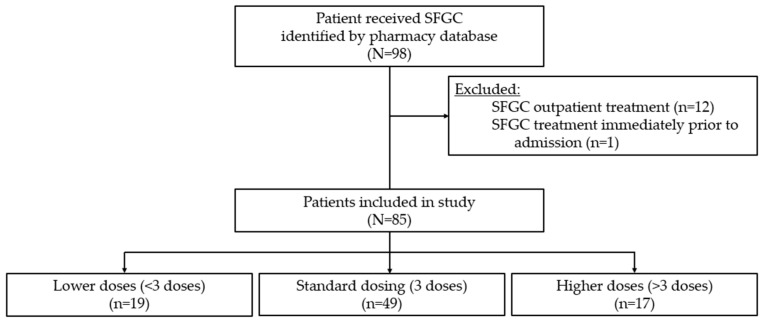
Study flow chart. Patient identification, inclusion, exclusion, and grouping by number of doses of SFGC received. SFGC: sodium ferric gluconate complex.

A total of 98 pediatric patients received SFGC for treatment of iron deficiency between 2016 and 2018 at our institution. This study aims to investigate the population of pediatric patients admitted to the hospital, so patients were excluded from the study if they received SFGC as an outpatient (n = 12) and if they were already receiving scheduled SFGC with hemodialysis sessions prior to their hospital admission (n = 1). A total of 85 patients were included in this study as demonstrated by Figure 2.

A total of 258 doses of SFGC were given to 85 patients at HDVCH between 2016 and 2018. As demonstrated in Table 1, the median patient age at admission was 49 months, with an interquartile range [IQR] of 9 to 158 months. The median patient weight at admission was 16.5 kg, with an IQR of 7.2 to 37.8 kg. Seventy-five (88.2%) of the patients were non-Hispanic, and 10 (11.8%) were Hispanic. Thirty-eight (44.7%) of the patients were male and 47 (55.3%) were female. The etiology of iron deficiency anemia and indications for SFGC therapy were reported based on the documented medical decision-making process at the time of infusion; 27.1% of patients experienced acute blood loss, 7.1% chronic blood loss, 32.9% malabsorption/nutritional deficiency, and 32.9% bone marrow suppression. The administration of epoetin during hospital stay was recorded as a binary variable (administration within 48 h before or after SFGC infusions), and 48.2% of patients received epoetin.

### 3.2. Patient Descriptions

Patients were more frequently admitted to the pediatric intensive care unit (63.5%) as compared to a general pediatric hospital admission (36.4%). PRISM III scores were calculated for the 54 patients admitted to the PICU with a median score of 5.0 [1.0, 10.0], which falls in the range of low risk of mortality without any statistically significant difference between the various dosing groups. There were two patients that scored 21 and 33, which is in the high risk of mortality category. Blood loss was recorded for 60 out of the 85 patients, with a median of 25.6 [5.8, 112.6] mL of blood loss. The time from admission to first dose of SFGC administration was a median of 90.7 [36.0, 214.0] hours. The length of stay was a median of 13.0 [5.3, 24.7] days. There was statistically significant evidence to suggest a difference in distribution of length of stay between the dose groups, and more specifically those with >3 doses had over double the length of stay (24.5 days for greater than three dose cohort versus 10.4 for equal to three dose cohort versus 9.3 days for less than three dose cohort).

### 3.3. Frequency and Distribution of Dosing

**Figure 3:**
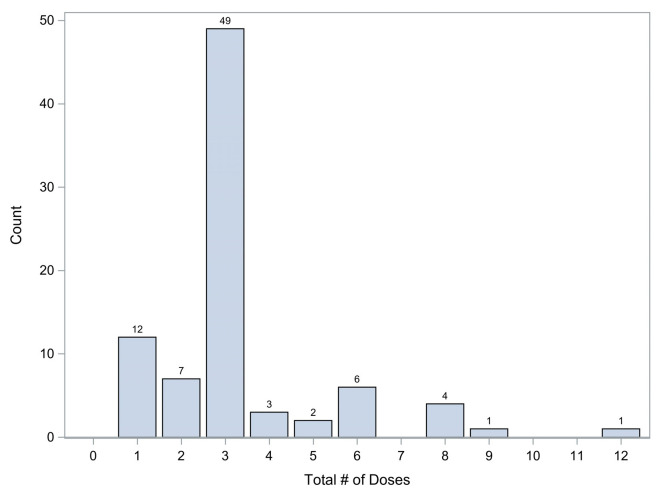
Frequency distribution of dosing. The frequency of patients who received each total number of sodium ferric gluconate complex doses.

The majority of patients, 49 (57.6%), received three doses of SFGC given once daily for three consecutive days in accordance with the current dosing protocol at HDVCH. While 19 (22.3%) received less than three doses and 17 (20%) received greater than three doses. All patients received SFGC doses of 1.5–2 mg/kg. The patients who received less than our institutional standard of a three-dose series of SFGC either did not have PBMP involvement or did have the recommendation for a three-dose series from PBMP, however changes in their care such as discharge from the hospital resulted in not completing the series. The patients who received more than three doses had complex and evolving clinical situations that led to perceived need for repeated series, usually based on repeated low iron indices in the face of continued anemia or subacute blood loss. Figure 3 demonstrates the frequency distribution of dosing in our study population.

### 3.4. Description of Hematologic Value Changes

Table 2 provides a description of the changes in serum laboratory values pre-treatment versus 7 and 14 days post-treatment with SFGC. The average pre-treatment serum hemoglobin was 8.73 ± 1.33; 7 days post-treatment it increased to 10.41 ± 1.43; and 14 days post-treatment increased to 10.76 ± 1.59. Mean corpuscular volume, ferritin, serum ion, total iron binding capacity, CRP, reticulocyte percentage, and reticulocyte hemoglobin all increased (except in the case of CRP which decreased) 7 and 14 days post-treatment as would be suspected.

### 3.5. Hematologic Values and Cumulative Dose Correlations

**Figure 4:**
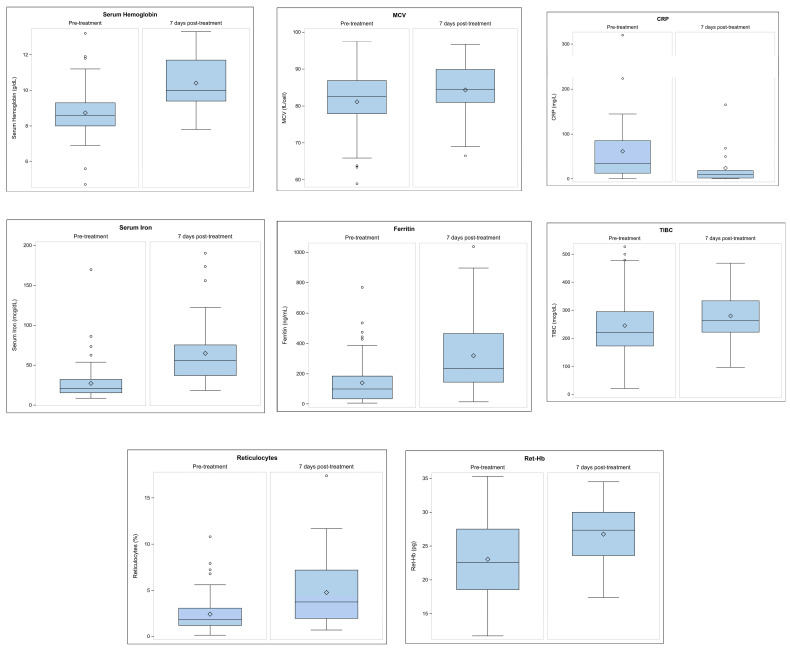
Pre-treatment versus 7 days post-treatment hematologic laboratory values for cumulative dose of SFGC received. This figure demonstrates the change in hematologic outcome variables from pre-treatment to 7 days post-treatment based on cumulative dose of sodium ferric gluconate complex received. Data from 14 days post-treatment were excluded due to high burden of missing data points and statistical insignificance. SFGC: sodium ferric gluconate complex, MCV: mean corpuscular volume, TIBC: total iron binding capacity, CRP: c-reactive protein. ° Circles in the boxplots represent outliers. An outlier is described as an observation that is at least 1.5 times the interquartile range (IQR) less than quartile 1 or at least 1.5 times the IQR more than quartile 3.

To further investigate hematologic values, we utilized the exposure variable, SFGC, as a continuous variable to determine if the amount of SFGC received was correlated with a significant change in hematologic values 7 days post-treatment as demonstrated by Table 3 and Figure 4. We found no correlation between cumulative dose of SFGC and serum hemoglobin, ferritin, serum iron, TIBC, CRP, reticulocytes, or reticulocyte hemoglobin. There was a mild-to-moderate positive correlation between cumulative dose and change in MCV from pre-treatment to 7 days post-treatment.

### 3.6. Impact on RBC Transfusions

A total of 51.8% of patients received RBC transfusions, with a median of 231 [76, 403] mL administered for all patients. Table 4 demonstrates these data for all three dosing cohorts. There is no statistically significant difference observed between SFGC dosing cohorts.

We utilized the exposure variable, SFGC, as a continuous variable to determine if the amount of SFGC received was correlated with the amount of RBC transfused (mL), and there was no statistically significant correlation observed as demonstrated in Table 5.

### 3.7. Safety

There was one adverse event recorded within our cohort. A fever was noted in a patient while receiving their fifth dose of SFGC during their admission. Of note, the patient had intermittently been febrile in the days prior to this event and had a confirmed viral infection with suspected endocarditis at that time. No severe adverse reactions, defined as hypotension, tachycardia, and need for intervention were noted in the study.

## 4. Discussion

Iron deficiency and anemia are common findings in pediatric patients [1,2,3]. Considering the well-established recommendation for restrictive RBC transfusion strategies [7], IV iron must be considered. This is especially impactful for the hospitalized pediatric patient population where enteral iron is not always a feasible alternative; for example, in patients with feeding intolerance or frequent NPO periods for procedural interventions. Iron sucrose and SFGC are rapid release IV iron preparations. There are more data surrounding the use of iron sucrose in pediatrics, including a recent prospective publication by Butragueno-Laiseca L et al. demonstrating the safety and efficacy of iron sucrose in the critically ill pediatric population [17]. However, SFGC label indication is for patients 6 years and older with chronic kidney disease undergoing hemodialysis in conjunction with supplemental erythropoietin therapy, so further data investigating its use in the hospitalized child are limited and retrospective.

Hassan et al. [20] retrospectively reviewed the use of SFGC in hospitalized pediatric patients with anemia due to iron deficiency or acute blood loss at our institution over a 7-year period, prior to the initiation of our study. They evaluated the efficacy of SFGC by comparing hematologic values pre-treatment and 2–4 days post-treatment and concluded a statistically significant increase in iron saturation, ferritin, reticulocyte count, and hemoglobin. Most infusion courses were accompanied by epoetin administration and demonstrated an even more robust response in hematologic values. However, this study excluded any infusion courses associated with RBC transfusions. These findings complement our conclusion that less than or equal to three doses of SFGC can produce an adequate hematologic response in the hospitalized pediatric population, and our study sought to build upon these findings by investigating the impacts of cumulative dosing of SFGC and the correlation with RBC transfusions.

Our data demonstrated increases in serum hemoglobin, MCV, ferritin, serum iron, TIBC, reticulocytes, and reticulocyte hemoglobin, with anticipated decrease in CRP following therapy with varying doses of SFGC infusions. However, we found no statistically significant correlation with cumulative dosing of SFGC, or statistically significant differences amongst the various dosing groups (less than three, equal to three, or greater than three doses) of SFGC. We also found no statistically significant correlation with cumulative dosing of SFGC on the amount of transfused RBC received. These findings suggest that there is no benefit on hematologic outcomes or the amount of transfused RBCs by providing the higher cumulative doses of SFGC to the hospitalized pediatric population, which is not what we hypothesized.

It is interesting that the patients who received greater than three doses of SFGC during their admission seemingly had acute blood loss as the indication for SFGC, more frequently trended towards receiving more transfused blood, more likely had a PICU admission, and had a longer hospitalization length of stay which all likely represent a covariate for severity of illness. Despite the consideration of these patients being more severely ill, there was also a notable latency between the first dose of SFGC in these patients who received greater than three doses of SFGC. This latency may be due to the increased complexity and severity of illness of these patients, which delayed either the development, diagnosis, or treatment of their iron deficiency anemia. Clinically, these patients were also more likely to receive erythropoietin, without any observed difference in bone marrow suppression, which likely implies the treatment intention was to timely improve red cell volume to avoid transfusion and/or the complications of anemia. Thus, it seems as though the patients who received a higher cumulative dosing of SFGC were significantly different than the rest of the study patients and required significantly different treatments which needs to be studied further. The appendix further characterizes this cohort by admission diagnosis, and hematologic outcomes by dosing group [Appendix A and Appendix B].

There is strength in this study’s contribution to the very limited data regarding SFGC use in the hospitalized pediatric population. We have provided data on multiple hematologic values for various dosing regimens, as well as the impact on RBC transfusion amounts. While we did not detect any statistically significant differences, the descriptions and data provided for the lower versus higher dosing are clinically relevant. We also demonstrated the safety of this IV iron formulation in this population, as we only observed one potential adverse event.

There are limitations to this study including the retrospective nature of the design and missing data points. The secondary hematologic outcome variables demonstrated the highest burden of missing data points, which is likely due to the HDVCH Blood Management Program’s monitoring pathway which includes repeat serum analyses recommended for 7 or 14 days post-treatment, depending on individual patient needs. Often, patients were discharged prior to these data being collected. Common to retrospective observational studies, we may have residual confounders. Numerous factors not investigated independently likely contributed to the patient’s dynamic clinical range, including analysis of comorbidities, dosing information regarding epoetin, and inaccurate recording of blood loss. The lack of these assessments, as well as the small sample size limits the generalization of the results observed in patients who received epoetin or had significant blood loss.

## 5. Conclusions

Our results encourage the trend of increased use of IV iron in the pediatric population as part of a restrictive RBC transfusion strategy, and the use of SFGC administered as a 1.5–2 mg/kg dose once daily for three consecutive days to achieve desired hematologic outcomes via changes in serum hemoglobin. With only one reported adverse event, our study results confirm the emerging evidence of favorable safety profiles of IV iron in the pediatric population [18,19,27,28,29]. Additional research is needed to describe the impact of SFGC dosing and resultant hematologic outcomes in the pediatric population with iron deficiency who experience ongoing blood loss, require longer lengths of stay, and have more complex diagnoses since the benefit of multiple courses of SFGC remains ambiguous.

## Figures and Tables

**Table 1:** Descriptive characteristics and outcomes by SFGC dose cohort.

Characteristic/Outcome	Overall (N = 85)	<3 Doses (n = 19)	3 Doses (n = 49)	>3 Doses (n = 17)	*p*-Values
Age, months	49 [9, 158]	108 [12, 185]	46 [8, 147]	47 [7, 123]	0.2584
Gender, male	38 (44.7)	4 (21.1)	26 (53.1)	8 (47.06)	0.0572
Weight, kg	16.5 [7.2, 37.8]	17.9 [8.3, 48.3]	15.8 [7.0, 36.6]	17.5 [7.1, 30.3]	0.6246
Ethnicity					1.0000
Non-Hispanic/Unknown	75 (88.2)	17 (89.5)	43 (87.8)	15 (88.2)	
Hispanic	10 (11.8)	2 (10.5)	6 (12.2)	2 (11.8)	
Admission					0.17931
General Pediatrics	31 (36.4)	10 (52.6)	17 (34.7)	4 (23.5)	
Pediatric Intensive Care Unit	54 (63.5)	9 (47.4)	32 (65.3)	13 (76.5)	
PRISM III Score *	5.0 [1.0, 10.0]	5.0 [5.0, 9.0]	4.0 [0.0, 10.0]	5.0 [1.0, 14.0]	0.8317
		n = 9	n = 31	n = 13	
Indications for SFGC					0.0817
Acute Blood Loss	23 (27.1)	3 (15.8)	12 (24.5)	9 (52.9)	
Chronic Blood Loss	6 (7.1)	0 (0)	5 (10.2)	0 (0)	
Malabsorption/Nutritional	28 (32.9)	6 (31.6)	7 (14.3)	1 (5.9)	
Bone Marrow Suppression	28 (32.9)	10 (62.6)	25 (51.0)	7 (41.2)	
Received Epoetin	41 (48.2)	7 (36.8)	23 (46.9)	11 (64.7)	0.2384
Length of Stay, days	13.0 [5.3, 24.7]	9.3 [3.0, 20.8]	10.4 [5.2, 20.8]	24.5 [20.0, 35.7]	0.0006
Time from Admission to first Dose, hours	90.7 [36.0, 214.0]	73.3 [25.0, 223.5]	67.1 [32.7, 182.8]	197.8 [91.8, 311.6]	0.0711
Total Blood Loss, mL	25.6 [5.8, 112.6]	27.5 [8.0, 100.5]	25.0 [6.5, 78.8]	30.0 [4.5, 144.0]	0.8682
	N = 60	n = 10	n = 33	n = 17	

Characteristics and outcome data for all patients provided and specified based on those who received <3 doses, 3 doses, and >3 doses of SFGC. SFGC: sodium ferric gluconate complex. * Patients admitted to the pediatric intensive care unit only.

**Table 2:** Hematologic laboratory descriptions for pre-treatment versus 7- and 14-day post-treatment with SFGC.

Variable	Pre-Treatment (N = 85) [Mean ± SD, (n)]	7 Days Post-Treatment (N = 85) [Mean ± SD, (n)]	14 Days Post-Treatment (N = 85) [Mean ± SD, (n)]
Serum Hemoglobin, g/dL	8.73 ± 1.33 (81)	10.41 ± 1.43 (47)	10.76 ± 1.59 (40)
MCV, fL	81.16 ± 7.56 (81)	84.32 ± 7.08 (47)	83.65 ± 7.58 (40)
Ferritin, mcg/L	140.47 ± 144.62 (76)	319.84 ± 242.04 (39)	356.19 ± 349.66 (16)
Serum Iron, mcg/dL	23.94 ± 22.23 (78)	62.35 ± 42.86 (37)	70.35 ± 36.04 (17)
TIBC, mcg/dL	245.32 ± 111.02 (78)	280.16 ± 84.26 (38)	292.65 ± 75.67 (17)
CRP, mg/L	61.66 ± 69.40 (34)	24.09 ± 43.62 (15)	11.13 ± 16.74 (20)
Reticulocytes, %	2.43 ± 1.87 (77)	4.77 ± 3.59 (39)	1.95 ± 1.13 (13)
Reticulocyte Hgb, pg	23.05 ± 5.63 (76)	26.75 ± 4.37 (36)	28.32 ± 5.81 (13)

These descriptive data demonstrate the observed change in hematologic outcomes variables from pre-treatment to 7 and 14 days post-treatment with sodium ferric gluconate complex for all patients. This represents the whole sample, not considering how many doses or the cumulative dose of SFGC received. SFGC: sodium ferric gluconate complex, MCV: mean corpuscular volume, TIBC: total iron binding capacity, CRP: c-reactive protein.

**Table 3:** Cumulative dose correlations for hematologic laboratory values.

Variable	Pearsons Correlation Coefficient	*p*-Value
Serum Hemoglobin, g/dL	−0.17781	0.2482
MCV, fL	0.33262	0.0256
Ferritin, mcg/L	0.08861	0.6127
Serum Iron, mcg/dL	−0.04321	0.8083
TIBC, mcg/dL	−0.19297	0.2667
CRP, mg/L	−0.00158	0.9963
Reticulocytes, %	0.07438	0.6527
Reticulocyte Hemoglobin, pg	0.25553	0.1325

This table demonstrates the cumulative dose of sodium ferric gluconate complex correlation to the change in hematologic outcome variables from pre-treatment to 7 days post-treatment. No correlation was found. Data from 14 days post-treatment were excluded due to high burden of missing data points and statistical insignificance. MCV: mean corpuscular volume, TIBC: total iron binding capacity, CRP: c-reactive protein.

**Table 4:** Descriptive RBC transfusion characteristics by SFGC dose cohort.

Characteristic	Overall (N = 85)	<3 Doses (n = 19)	3 Doses (n = 49)	>3 Doses (n = 17)	*p*-Values
Patients who received RBC, number (%)	44 (51.8)	6 (31.6)	28 (57.1)	10 (58.8)	0.1348
Total RBC transfused, mL, Median [IQR]	231 [76, 403]	325 [236, 494]	159 [62, 305]	372 [173, 530]	0.1221

Characteristic data relating to RBC transfusion for all patients provided and specified based on those who received <3 doses, 3 doses, versus >3 doses of SFGC. RBC: red blood cell, SFGC: sodium ferric gluconate complex.

**Table 5:** Cumulative dose correlation for RBC volume.

Variable	Pearsons Correlation Coefficient	*p*-Value
RBC Transfusion Received, mL × Cumulative dose SFGC	0.17511	0.2556

This table demonstrates the cumulative dose of sodium ferric gluconate complex correlation to the amount of RBC transfusions received. No correlation was found. RBC: red blood cell, SFGC: sodium ferric gluconate complex.

## Data Availability

The raw data supporting the conclusions of this article will be made available by the authors on request. The data are not publicly available due to technical and time limitations.

## Abbreviations

CPCCRN: Collaborative Pediatric Critical Care Research Network; CRP: c-reactive protein; EPO: recombinant erythropoietin; IV: intravenous; MCV: mean corpuscular volume; PICU: pediatric intensive care unit; PRISM: Pediatric Risk of Mortality; RBC: red blood cells; SFGC: sodium ferric gluconate complex; TIBC: total iron binding capacity.

## Appendix A

**Table A1:** Hematologic laboratory descriptions for pre-treatment versus 7 and 14 days post-treatment per SFGC dose cohort.

Variable	Post Infusion	<3 Doses Change from Baseline (n = 19) [Mean ± SD, (n)]	3 Doses Change from Baseline (n = 49) [mean ± SD, (n)]	>3 Doses Change from Baseline (n = 17) [Mean ± SD, (n)]	*p*-Value
Hemoglobin, g/dL	7d	1.81 ± 1.26 (9)	1.81 ± 2.06 (25)	1.51 ± 1.64 (10)	0.9031
	14d	2.89 ± 2.59 (9)	1.33 ± 1.58 (20)	1.91 ± 1.34 (7)	0.1226
Mean Corpuscular Volume, fL	7d	1.98 ± 4.71 (10)	2.77 ± 4.70 (25)	4.22 ± 3.01 (10)	0.5117
	14d	0.81 ± 4.03 (9)	2.33 ± 4.58 (20)	0.99 ± 2.69 (7)	0.5900
Ferritin, mcg/L	7d	293.2 ± 278.2 (5)	127.2 ± 110.0 (23)	80.85 ± 168.95 (7)	0.0580
	14d	35.5 ± 108.19 (2)	142.78 ± 194.18 (9)	416.5 ± 460.3 (2)	NA
Serum Iron, mcg/dL	7d	66.40 ± 70.83 (5)	31.50 ± 42.22 (22)	41.0 ± 37.8 (7)	0.3220
	14d	23.00 ± 42.25 (4)	23.86 ± 79.58 (7)	33.0 ± 0 (2)	NA
TIBC, mcg/dL	7d	96.00 ± 70.65 (5)	49.13 ± 64.54 (23)	73.29 ± 94.67 (7)	0.3763
	14d	56.25 ± 73.45 (4)	105.14 ± 79.20 (7)	93.00 ± 80.61 (2)	NA
CRP, mg/L	7d	NA	−72.46 ± 70.51 (8)	−198.10± 101.40 (2)	NA
	14d	−10.70 ± 47.23 (2)	−58.94 ± 64.80 (9)	NA	NA
Reticulocyte, %	7d	1.01 ± 2.82 (6)	2.77 ± 3.78 (25)	2.20 ± 3.41 (8)	0.5583
	14d	0.84 ± 0.29 (3)	0.34 ± 1.32 (8)	−0.46 ± 1.87 (2)	NA
Reticulocyte Hgb, pg	7d	4.42 ± 4.54 (6)	2.75 ± 6.66 (22)	2.96 ± 3.54 (8)	0.8252
	14d	0.30 ± 8.49 (3)	2.14 ± 5.01 (8)	3.95 ± 8.41 (2)	NA

These descriptive data demonstrate the observed change in hematologic outcomes variables from pre-treatment to 7 and 14 days post-treatment with sodium ferric gluconate complex for patients specified based on those who received <3 doses, 3 doses, and >3 doses of SFGC. SFGC: sodium ferric gluconate complex, TIBC: total iron binding capacity, CRP: c-reactive protein, NA: sample size was too small to calculate or report.

## Appendix B

**Table A2:** Characterization of the >3 dose group by admission diagnosis.

Number of Doses of Sodium Ferric Gluconate Received (n)	Admission Diagnosis
4	Hematogenous Osteomyelitis of Left Femur
4	Status-Post Right Hemicolectomy
4	Ulcerative Colitis with Rectal Bleeding
5	Parapneumonic Effusion
5	Bronchiolitis, Failure to Thrive
6	Gastrointestinal Bleed
6	Partial Traumatic Amputation of Left Foot
6	Acute Respiratory Failure, Cardiac Arrest, Cranial Synostosis
6	Hydrocephalus: ETV/CPC Procedure Failure
6	Truncus Arteriosus
6	Grade IV Liver Laceration
8	Acute Respiratory Failure with Hypoxia, Toxic Shock Syndrome
8	Trauma, Traumatic Brain Injury, Motor Vehicle Collision
8	Acute Respiratory Failure with Hypoxia and Hypercapnia
8	Pericardial Effusion, Pulmonary Stenosis, Noonan Syndrome
9	Cerebritis Status Post Craniotomy
12	Shiga Toxic Hemolytic Uremic Syndrome

A total of 17 patients received >3 doses of sodium ferric gluconate complex. This table further describes this dose group by demonstrating the number of doses received by each patient, as well as their diagnosis on admission. ETV: endoscopic third ventriculostomy, CPC: choroid plexus cauterization.
